# Chromosome-scale scaffolding of the black raspberry (*Rubus occidentalis* L.) genome based on chromatin interaction data

**DOI:** 10.1038/s41438-017-0013-y

**Published:** 2018-02-07

**Authors:** Rubina Jibran, Helge Dzierzon, Nahla Bassil, Jill M. Bushakra, Patrick P. Edger, Shawn Sullivan, Chad E. Finn, Michael Dossett, Kelly J. Vining, Robert VanBuren, Todd C. Mockler, Ivan Liachko, Kevin M. Davies, Toshi M. Foster, David Chagné

**Affiliations:** 1 The New Zealand Institute for Plant & Food Research Limited, Private Bag 11600, Palmerston North, 4474 New Zealand; 2 USDA-ARS National Clonal Germplasm Repository, 33447 Peoria Road, Corvallis, OR 97333 USA; 3 Department of Horticulture, Michigan State University, East Lansing, MI 48824-2604 USA; 4 Phase Genomics, Seattle, WA 98195 USA; 5 USDA-ARS Horticultural Crops Research Unit, Corvallis, OR 97330 USA; 6 B.C. Blueberry Council (in Partnership with Agriculture and Agri-Food Canada) Agassiz Food Research Centre, Agassiz, BC V0M 1A0 Canada; 7 Department of Horticulture, Michigan State University, East Lansing, MI 48824-2604 USA; 8 The Donald Danforth Plant Science Center, St. Louis, MO 63132 USA; 9 Department of Genome Sciences, University of Washington School of Medicine, Seattle, WA 98195 USA; 10 The New Zealand Institute for Plant & Food Research Limited, Private Bag 11600, Palmerston North, 4474 New Zealand

## Abstract

Black raspberry (*Rubus occidentalis* L.) is a niche fruit crop valued for its flavor and potential health benefits. The improvement of fruit and cane characteristics via molecular breeding technologies has been hindered by the lack of a high-quality reference genome. The recently released draft genome for black raspberry (ORUS 4115-3) lacks assembly of scaffolds to chromosome scale. We used high-throughput chromatin conformation capture (Hi-C) and Proximity-Guided Assembly (PGA) to cluster and order 9650 out of 11,936 contigs of this draft genome assembly into seven pseudo-chromosomes. The seven pseudo-chromosomes cover ~97.2% of the total contig length (~223.8 Mb). Locating existing genetic markers on the physical map resolved multiple discrepancies in marker order on the genetic map. Centromeric regions were inferred from recombination frequencies of genetic markers, alignment of 303 bp centromeric sequence with the PGA, and heat map showing the physical contact matrix over the entire genome. We demonstrate a high degree of synteny between each of the seven chromosomes of black raspberry and a high-quality reference genome for strawberry (*Fragaria vesca* L.) assembled using only PacBio long-read sequences. We conclude that PGA is a cost-effective and rapid method of generating chromosome-scale assemblies from Illumina short-read sequencing data.

## Plant genomics: Improved genome for the black raspberry

Assembly of a high-quality reference genome for the black raspberry plant will inform future crop improvements. Sequencing the genomes of widely-grown, profitable fruit crops can help researchers identify the DNA markers linked to desirable traits, potentially improving crop health and yields. David Chagné at the New Zealand Institute for Plant and Food Research Limited and co-workers combined new techniques to significantly improve on an existing genome for the black raspberry (Rubus occidentalis L.). The researchers used Hi-C analysis to create a map identifying the interactions between chromatin fragments – macromolecules made from DNA, proteins and RNA – and the three-dimensional structure of chromosomes inside the cell nucleus. Then, they used a bioinformatics assembly method to construct the genome from this data. The new genome showed high accuracy when compared to another genome from the same family.

## Introduction

Despite recent advances in DNA sequencing technologies and computational approaches, the de novo assembly of high-quality reference genomes in plant species using solely short-read sequencing data remains difficult. One of the biggest challenges is scaffolding contigs into chromosome-scale sequences, as construction of short gun libraries with large inserts (>15 kb) is extremely difficult and de novo assembly algorithms tend to not perform well across repetitive sequences. Chromosome-scale reference genomes have only been obtained for a few selected horticultural species including strawberry^1^, peach^2^, and apple^3^ largely with the aid of available genetic maps to anchor scaffolds.

Several methods can be used for scaffolding. Bacterial artificial chromosome (BAC)-end sequencing is one approach; however, it is expensive, tedious and time consuming. High-density genetic maps can be used for scaffolding; however, it is often difficult to obtain sufficient numbers of markers to anchor and orientate small contigs and the mapping algorithms used to build genetic maps can sometimes place markers at incorrect locations^4^. Furthermore, there is not a direct relationship between centiMorgan (cM) on linkage maps, which is a measure of recombination frequency, and physical distance expressed as megabases (Mb) of sequence data.

De novo scaffolding has been demonstrated in both haploid and diploid organisms using chromosome conformation capture (3C) followed by Hi-C analysis^5,6^. Hi-C is a novel strategy combining capture of chromatin interaction within the nucleus and next-generation sequencing (NGS). New bioinformatics methods such as proximity-guided assembly (PGA) can be used for developing near-complete pseudo-chromosome assemblies of complex genomes using Hi-C data. Hi-C relies on the folding of chromosomes inside the nucleus so that fragments sequenced from the same chromosome are in close three-dimensional proximity, whereas fragments from different chromosomes are more distant. The method involves an initial chemical cross-linking of chromatin to capture chromosome fragments that are in close physical proximity, digestion of the captured fragments with restriction enzymes to create cuts close to the fixed area, ligating the cut ends and then sequencing the junctions using NGS^7^. A major benefit of this method is that the cross-linking process is random and independent of DNA sequence. The probability of finding fragments that were in close proximity on the same chromosome within paired-end NGS reads decreases with physical distance and decreases even more drastically between fragments from different chromosomes. This method has been used in prokaryotes and some eukaryotes, including humans, to dramatically improve genome assemblies, infer the location of previously unanchored contigs, and create chromosome length haplotypes^5,8,9^.

Because of their economic importance, several members of the Rosaceae family have fully or partially assembled genomes. The genomes of apple (*Malus* × *domestica* Borkh.), peach (*Prunus persica* [L.] Batsch) and diploid strawberry (*Fragaria vesca* L.) have been assembled as pseudo-chromosomes^1,3,10,11^. Asian and European pear (*Pyrus pyrifolia* [Burm.] Nak. and *P. communis* L., respectively), Chinese plum (*Prunus mume* Siebold & Zucc.), and China rose (*Rosa chinensis* Jacq.) all have partially anchored draft genomes^12–15^. Diploid black raspberry (*Rubus occidentalis* L.) has been recently sequenced and assembled into draft genome spanning 82% of the genome^16^. There are 11,936 black raspberry scaffolds and the scaffold N50 length is 49,488 bp. We performed Hi-C sequencing of the same genotype (ORUS 4115-3) sequenced by VanBuren et al.^16^ to order these scaffolds into pseudo-chromosomes.

## Methods

### Genome sequence

The assembled genome of ORUS 4115-3 (Rubus_occidentalis_v1.0.a1) was downloaded from the Genomic Database for Rosaceae (www.rosaceae.org)^16^. ORUS 4115-3 was a selection made in Corvallis, OR (USDA) from a population collected near Rich Mountain, SC (USA) and is clonally maintained by the USDA-ARS, National Clonal Germplasm Repository as PI 672644 (Germplasm Resources Information Network, 2016)^15^. The input assembly has 11,936 contigs of a total length of 230,199,469 bp and an N50 of 49,488 bp.

### Proximity-guided assembly

Hi-C scaffolding was performed by Phase Genomics Ltd. (Seattle, WA, USA) Proximo Hi-C genome scaffolding service. A Hi-C library was prepared from 0.2 g of fresh *R. occidentalis* ORUS 4115-3 leaf tissue^17^. The Hi-C library was sequenced on the Illumina NextSeq platform (Illumina, La Jolla, CA, USA), generating 54.4 million Hi-C read pairs, which were provided as input to the Proximo Hi-C scaffolding pipeline. The Hi-C reads were aligned to the draft assembly using the “bwa aln” algorithm^18^ with the “-n 0” option, requiring reads to map without any mismatches, as has been suggested^19^. After filtering erroneous mappings (mapQ = 0) on the alignments, 16,521,894 read pairs were discarded because they mapped to the same contig and were therefore uninformative for scaffolding purposes. In total 5,220,846 read pairs remained where one read mapped to one contig and the other mapped to another contig, and were used in the scaffolding pipeline, an average of 437 informative Hi-C read pairs. Only read pairs in which read 1 (R1) and read 2 (R2) aligned to different contigs are useful for Hi-C scaffolding, because these pairs gives information about the structure of the genome. Read pairs that either mapped to the same contig or did not map cleanly enough were discarded in clustering analysis.

The Proximo Hi-C scaffolding pipeline performed chromosome assignment and scaffold ordering and orientation on the contigs using the Hi-C data as described previously^5,6^. Proximo includes an enhanced version of the LACHESIS algorithm^19^ (http://shendurelab.github.io/LACHESIS/), along with a scaffold optimization algorithm, extra reports and QC steps. This method places contigs into chromosome groups based on the strength of their Hi-C interactions, and then arranges and orientates them into a linear order such that the amount of Hi-C interactions among adjacent and nearby contigs is maximized. The results of this process were optimized over the course of 40,000 independent scaffolding attempts, to maximize the number of Hi-C links within scaffolds and the log-likelihood of the chosen placement and orientation of a contig having generated the observed Hi-C data relative to alternatives.

The post-clustering heat maps constructed made by the Proximo Hi-C pipeline as one of the report steps. Once scaffolding was complete, the contigs were placed into a linear order by scaffold, and then by position on the scaffold. These *x*-/*y*-coordinates were used in the heat map. To develop the “by_length” heat map, larger contigs were given some extra space in the *x*–*y* plane by allowing multiple coordinates in a row. The Hi-C link density between each pair of contigs was recorded, using the *x*-/*y*-coordinates we identified above to order them. “Link density” is the number of Hi-C links between a given pair of contigs normalized for the number of restriction sites in those contigs. The link density was plotted across 3000 bins for each pseudomolecule with bin size varying by pseudomolecule length. The heat map was plotted with an R script that uses the link density and scaffold boundary data. This script is the same one published with LACHESIS, with minor modifications that do not affect the analysis or primary figure content (e.g., programmatic interface tweaks, slightly different figure title).

*Rubus occidentalis* gene models^16^ were mapped to the Rubus_occidentalis_v1.1 assembly by using the default pipeline FLO (https://github.com/wurmlab/flo), which is based on the University of California, Santa Cruz (UCSC)-liftOver Toolkit^20^.

### Physical ordering of genetic markers and comparison of the PGA black raspberry assembly with parental genetic maps

The genotyping by sequencing (GBS)-based single nucleotide polymorphism (SNP) markers (Supplementary Tables S2 and S3)^16^ and centromere sequences were mapped and aligned to the reference genome assembly of ORUS 4115-3^16^ using the Genomic Mapping and Alignment Program (GMAP) with default parameters (gmap/2016-06-09)^21^. In total, 244 (for ORUS 4153–1) and 224 (for ORUS 3021–2) SNP markers, evenly distributed across the 7 chromosomes, were chosen for the comparison of the parental genetic maps and the PGA guided assembly genome (Supplementary Tables S2 and S3). We chose to focus on GBS SNP-based markers so we could apply a more systematic method to retrieve the markers within the assembly contigs based on their flanking sequences. The selected markers from each linkage group were aligned against the 7 pseudo-chromosomes of Rubus_occidentalis_v1.0.pga using GMAP (Supplementary Figures S1–7).

### *Trans*:*cis* quantitation

In order to examine the distribution of local (*cis*) and between chromosomes interactions (*trans*) for each pseudo-chromosome in the genome, the Hi-C data were processed using the HiCUP mapping program (www.bioinformatics.babraham.ac.uk/projects/hicup/).

The HiCUP Binary Alignment Map (BAM) file along with a file of restriction enzyme Sau3AI restriction fragments was imported into SeqMonk https://www.bioinformatics.babraham.ac.uk/projects/seqmonk/. A suitable set of probes was generated using *Sau*3AI restriction fragments. Fifty consecutive *Sau*3AI probes were grouped together to identify significant interactions (BoxWhisker filter) in the larger genomic regions. The quantitation was adjusted to subtract the median *trans* percentage from each chromosome overall value (https://www.bioinformatics.babraham.ac.uk/projects/seqmonk/), to identify the location variation in *trans* percentage and to view the *trans*:*cis* ratio in a more reliable way. SeqMonk was used to calculate the *trans*:*cis* ratio of the Hi-C data. This method counts the numbers of ends involved in a *cis* or a *trans* interaction in each probe and then quantifies the probes with percentage of *trans* reads. The Hi-C probes were generated around *Sau*3AI digestion fragments. The raw counts of *trans*:*cis* ratios can be misleading as the probes located on small chromosome will have more *trans* interaction and the probes of a longer chromosome will have larger *cis* reads. The randomly ligated fragments will be picked up as *trans* interactions and the percentage of these interactions is directly proportional to chromosome length. Therefore the median *trans* percentage values were extracted from overall values.

### Comparative genomics

Syntenic dot plots were generated with SynMap^22^ using LAST for whole-genome comparisons. A minimum of five synteny genes with a maximum distance of 50 kb between adjacent genes was used to seed a syntenic block. Syntenic gene pairs were colored based on their *K*_s_ values in order to differentiate orthologous and out-paralog syntenic blocks. Circularized syntenic plots were constructed using Circos^23^ with each connecting line representing orthologous gene sequences.

## Results

### Proximity-guided assembly of *R. occidentalis* (ORUS 4115-3) genome

**Fig. 1:**
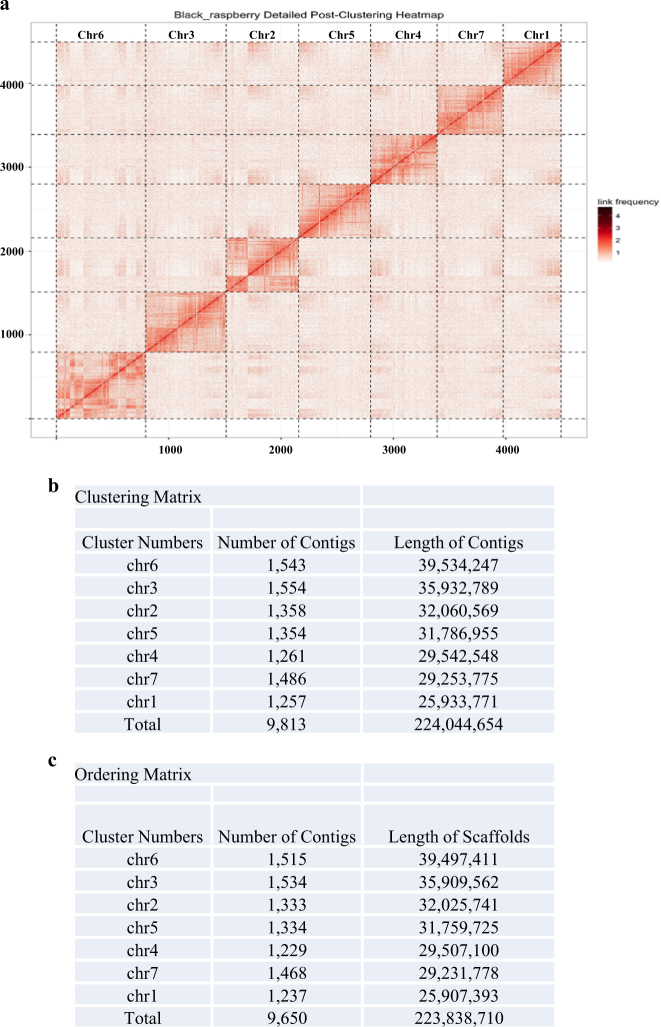
Chromatin conformation capture-based improved assembly of *Rubus occidentalis* genome of ORUS 4115-3 (Rubus_occidentalis_v1.0.pga). **a** Post-clustering heat map showing the density of Hi-C interactions between scaffolds used for proximity-guided assembly (PGA). Seven pseudo-chromosomes are arranged by size with the largest clusters sorted towards the bottom left of the matrix. The submatrix shown here corresponds to intrachromosomal and interchromosomal interactions in the genome. Each pixel represents all interactions between one 34.4 kb locus and another 34.4 kb locus and intensity corresponds to the total number of reads per interaction. Each rectangular box represents one chromosome. **b** Clustering of scaffolds using Hi-C data into pseudo-chromosome-scale scaffolds. Listed are the 9813 scaffolds of total length ~224 Mb used for clustering. Also listed in the table are the cluster numbers, the number of contigs, and the reference length of the contigs. **c** Scaffold ordering and orientation in the PGA clustering. Shown are the 9650 scaffolds of total length; ~223 MB were successfully ordered and orientated along the seven pseudo-molecules. Also listed in the table are the cluster numbers, the number of scaffolds in the derived ordering and the reference length of the pseudo-chromosomes

In total, 52.2 million Hi-C read pairs were aligned to the 11,936 *R*. *occidentalis* contigs spanning 230,199,469 bp (97.3% of total sequence length). By clustering, ordering, and orienting groups of contigs that were likely to derive from a common chromosome, seven pseudo-chromosomes were defined (Fig. 1a). Of the clustered 9813 contigs, 9650 were ordered and oriented, spanning a total of 223,838,710 bp (97.2% of total sequence length) (Fig. 1b, c). The shortest and longest assembled pseudo-molecules were chromosomes 1 and 6 with 25,907,393 and 39,497,411 bp, respectively. Half of the scaffolded pseudo-chromosomes were longer than 31,759,000 bp, representing a dramatic increase in the N50 49,488 bp obtained in the original assembly, prior to PGA^16^. An additional 163 contigs were clustered into a 205,944 bp scaffold. However they could not be placed into that scaffold’s linear order with sufficient confidence to warrant their inclusion, likely due to the small size of those contigs (average size: 1263 bp). Our new *R. occidentalis* assembled genome of ORUS 4115-3 obtained by PGA is now referred to as Rubus_occidentalis_v1.1 and is available at the Genome Database for Rosaceae (https://www.rosaceae.org/24).

We aligned the predicted *Rubus* gene annotations^16^ to the new Rubus_occidentalis_v1.1 genome by using the FLO pipeline with default parameters. Out of the 28,005 protein-coding genes, 27,541 (98.3%) were mapped successfully. The structural information of the aligned mRNA sequences is described in Supplementary Table S1A–G. Chromosomes 1 to 7 have 3101, 4094, 4134, 3677, 3872, 5167, and 3496 protein-coding genes, respectively.

### Comparison of the PGA black raspberry assembly with parental genetic maps

**Fig. 2:**
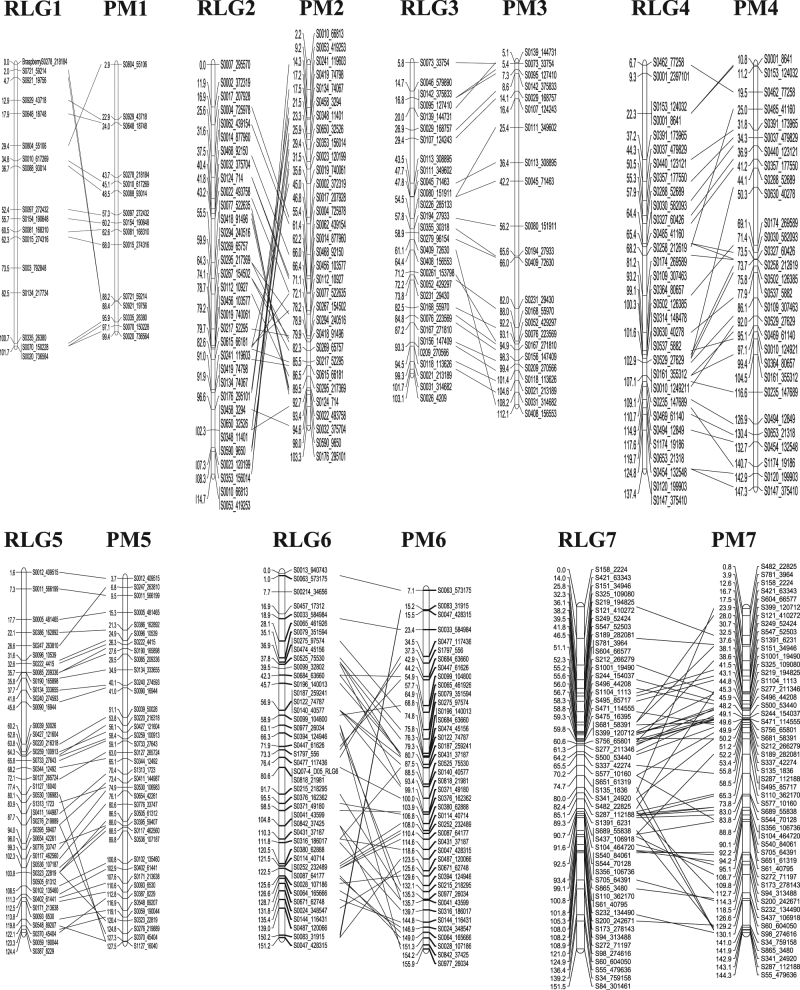
Comparison between the black raspberry genome assembly (Rubus_occidentalis_v1.0.pga) and genetic map of female parent ORUS 4153-115-3. The pseudo-chromosomes of Rubus_occidentalis_v1.0.pga (PM) are represented on the right and linkage group (LG) to the left. The names of the markers are shown to the right of each chromosome. The units to the left of each bar represent the physical and genetic locations of markers, respectively. The lines connect the physical positions of the markers with their genetic map positions. RLG Rubus linkage group, PM physical map of Rubus_occidentalis_v1.0.pga. The comparison was constructed using MapChart (http://www.joinmap.nl)

We performed a comparison between the order of markers in the parental linkage maps of ORUS 4153–1 x ORUS 3021–2 (Supplementary Tables S2 and S3)^16^ and the PGA guided genome assembly of Rubus_occidentalis_v1.1 to identify any conflicts arising from a shift in the genetic map position because of inaccuracies in recombination frequencies. The physical positions of 90% of ORUS 4153–1 and 78% of ORUS 3021–2 genetic markers were correctly identified in the Rubus_occidentalis_v1.0.pga genome. When the genetic map positions of the markers were compared with their physical orders (Fig. 2; Supplementary Figures S1–S8), the physical and genetic orders of ~85% of the markers were found to be consistent in both parents. However, there was inconsistency in the orders of 32 (13%) and 27 (12%) markers between the physical order and the ORUS 4153–1 and ORUS 3021–2 genetic maps, respectively (Table 1).

**Table 1:** Descriptive statistics of comparison between ORUS 4153–1 and ORUS 3021–2 genetic and physical maps

	No. of markers used for comparison	No. of markers identified in PGA	No. of consistent markers	No. of inconsistencies
ORUS 4153–1	244	224	192	32
ORUS 3021–2	224	176	149	27

The genotyping by sequencing (GBS)-based single nucleotide polymorphism (SNP) markers described by VanBuren et al.^16^ were mapped and aligned to the reference genome assembly of ORUS 4115-3^16^ and the new proximity-guided assembly

### Location of centromeres in the black raspberry genome

**Fig. 3:**
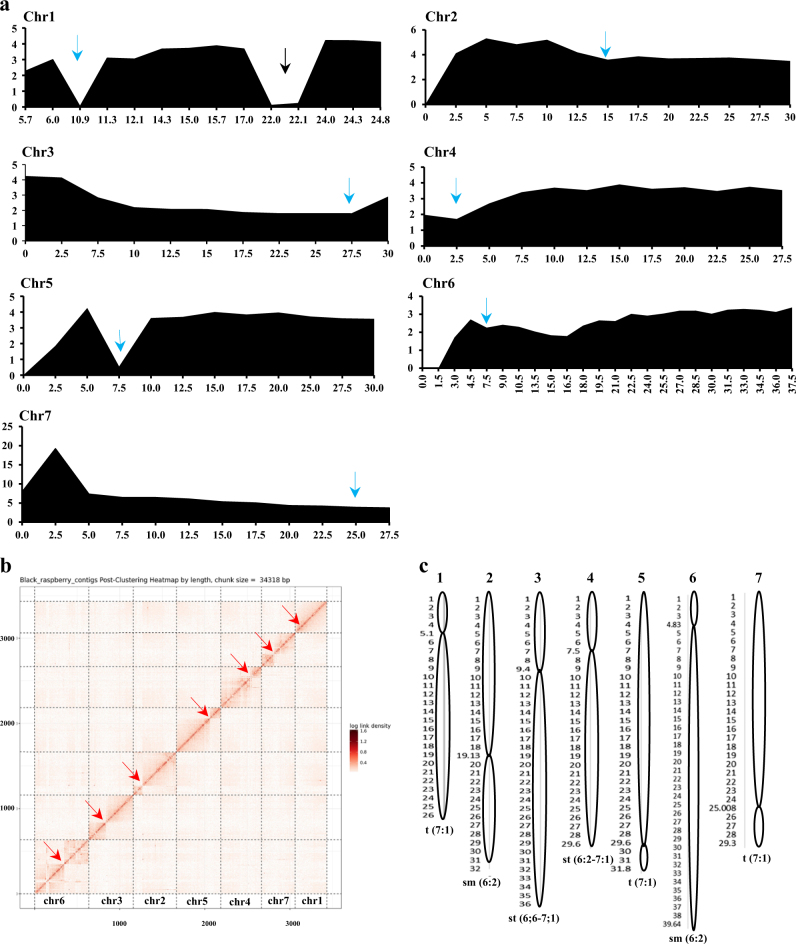
Identification of centromeres in the Rubus_occidentalis_v1.0.pga genome assembly. **a** Frequencies of genetic recombination in ORUS 4153–1 genome. The *x*-axis represents the physical distance in million base pairs (Mb) along the chromosomes. The *y*-axis represents the ratio of genetic distance to physical distance (cM/Mb) calculated from the map published by Bushakra et al.^24^ The average physical distance ranged from 2 to 4 Mb per cM across the chromosome, a dip in the recombination frequencies may correspond to centromere locations. The approximate centromere positions are marked with blue arrows. The black arrow represents a region of reduced recombination on chromosome 1. **b** Hi-C heat map showing the density of Hi-C interactions between scaffolds used for proximity-guided assembly. The seven pseudo-chromosomes are arranged by size with the largest clusters sorted towards the bottom left of the matrix. The submatrix shown here corresponds to intrachromosomal interactions in the genome. Each pixel represents all interactions between one 34.31 kb locus and another 34.31 kb locus and intensity corresponds to the total number of reads per interaction. The approximate centromere positions are marked with red arrow. **c** Physical maps of chromosomes showing *R. occidentalis* centromeric positions. Physical sizes of chromosomes were derived from Rubus_occidentalis_v1.1 assembly and chromosomes were classified by the method proposed by Naranjo et al.^38^ t terminal, sm submedian, st subterminal

The likely locations of centromeres in the black raspberry genome were inferred using three independent methods, and the results were largely consistent. First, the probability of crossovers in different regions of the genome was analyzed by comparing physical distances in million base pairs (Mb) with the genetic distances of the markers (Fig. 3a). The average physical distance ranged from 2 to 4 cM/Mb, which varied with position along and between the chromosomes. Eight regions of suppressed genetic recombination that are likely to be associated with the repetitive regions of the genome and centromeres were identified on the seven chromosomes (Fig. 3a). For example, clear reduced recombination frequencies were detected on chromosomes 1 and 5 at positions 10.9 and 7.5 Mb, respectively. On chromosome 7 the recombination frequency gradually decreased towards the end of the chromosome. Secondly, centromeric sequences were analyzed in the Rubus_occidentalis_v1.0.pga assembly, using the 303 bp conserved region described by VanBuren et al.^16^ (Supplementary Figure S9).

This conserved sequence was identified in all seven pseudo-chromosomes using GMAP. Finally, the Hi-C interaction data were used to validate the candidate positions of centromeres, as these regions are expected to display reduced intrachromosomal interactions in the Hi-C heat map (Fig. 3c). Figure 3a shows that such regions in the Hi-C heat map coincide with the candidate centromere locations identified by GMAP alignments and the recombination distribution patterns. For example, in chromosome 7 the reduced recombination frequency and the physical centromere location (identified by aligning 303 bp centromere sequence with the Hi-C assembly) were observed around position 25 Mb. However, some discrepancies were observed. For example the crossover events for chromosome 1 show two distinct dips at positions 10.9 and 22 Mb. Similarly, the recombination suppression is observed at position 7.5 Mb in chromosome 5, whereas the identified centromere location is around 30 Mb.

Chromosomes can be classified into one of six types based on the ratio of the long arm to the short arm^24^. Chromosomes 1, 5, 6, and 7 of black raspberry were classified as chromosomes with terminal (t) centromeres, chromosome 2 was classified as chromosome with submedian centromere (sm), and chromosomes 3 and 4 were classified as chromosomes with subterminal (st) centromeres (Fig. 3c).

### Interchromosomal and intrachromosomal interactions

**Fig. 4:**
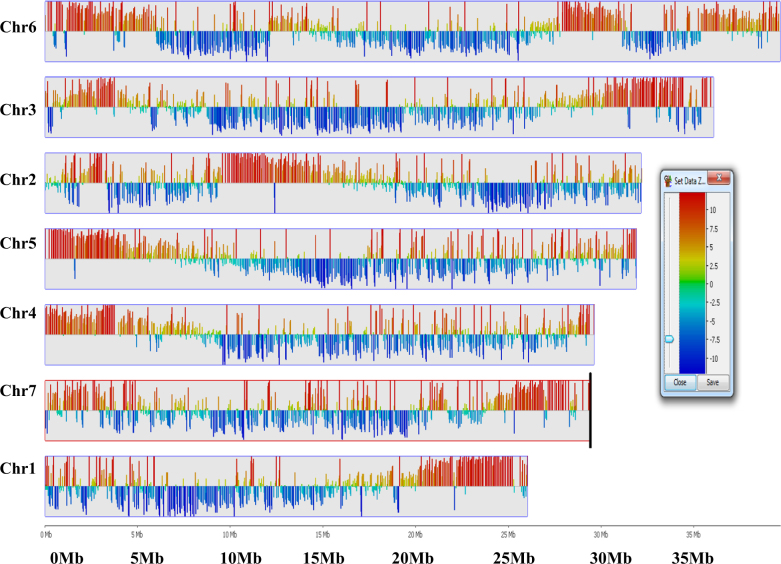
Distribution of *trans* percentages across the black raspberry genome. Red bars indicate widely interacting domains, blue bars indicate local interactions within the chromosome. To eliminate the artefacts in *trans*:*cis* calculations the extreme outliers were removed by using BoxWhisker filter (statistical test); *trans*:*cis* ratios were calculated in SeqMonk. The strength of the interaction is represented by colors of the scale bar showing low values with cool blue tones while higher values with hotter orange and red tones

*Trans*:*cis* ratios of Hi-C reads can be useful in determining genome regions with potential true *trans* and *cis* interactions. The distribution of corrected *trans* percentage across the genome calculated using SeqMonk (Fig. 4) clearly shows genomic regions that have either high or low *trans* percentage, indicating either distant or local interactions, respectively. The *trans*:*cis* ratio decreases when a genomic region strongly interacts with itself (represented by blue bars) and the ratio increases (represented by red bars) for long-range interchromosomal interactions. The information on the placement of these chromatin domains provides an insight into their general behaviors in the genome.

### Comparison of the PGA black raspberry assembly with *Fragaria vesca*

**Fig. 5:**
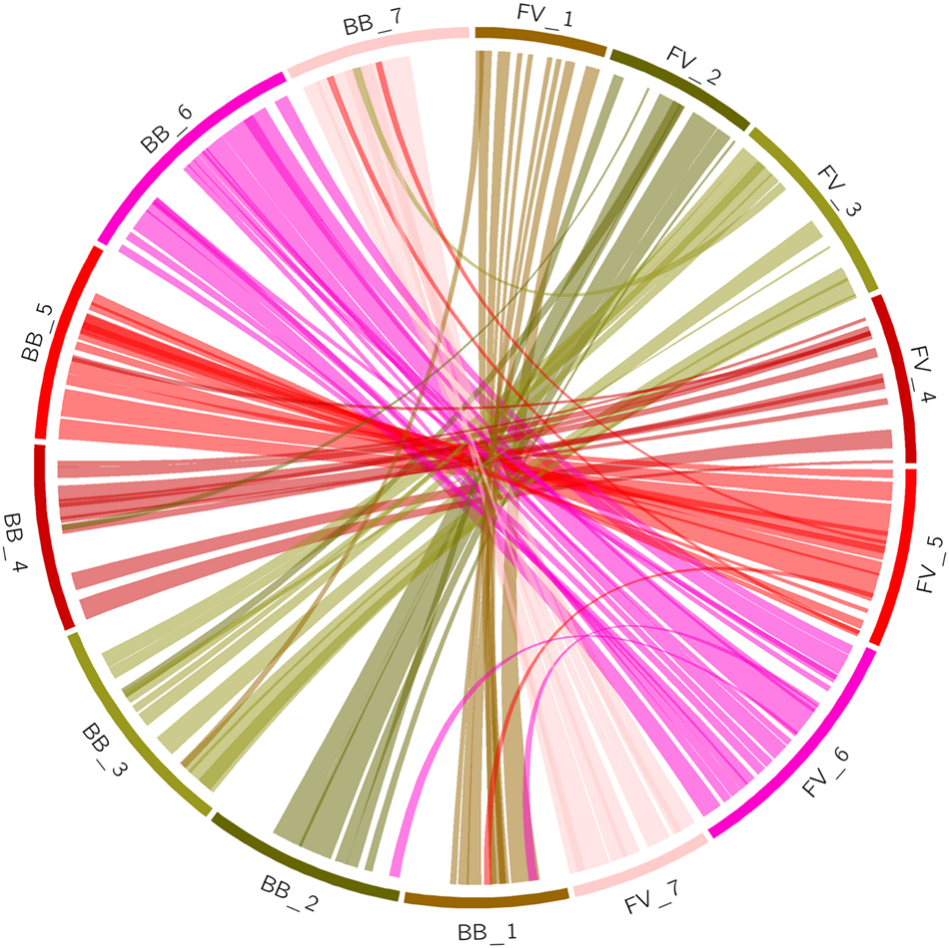
Circos plot showing macro-synteny between the black raspberry and strawberry genomes. Each connecting line represents a syntenic fragment of 50 kb. The figure uses the *Rubus* karyotype file which defines seven chromosomes (1–7) for black berry and strawberry. BB black berry, FV strawberry

Alignment of our new black raspberry genome with the high-quality diploid strawberry genome (*F. vesca* V4)^25^ showed that there is a high degree of synteny between these genomes. Figure 5 depicts their positional relationships with circularly arranged ideograms and illustrates that there are only a few potential small transversions between the genomes of black raspberry and strawberry.

## Discussion

We have assembled the genome of black raspberry ORUS 4115-3^16^ to the chromosome scale using the Hi-C technique followed by PGA. We have confirmed and resolved the positons of genetic markers and identified centromeres on each of the black raspberry chromosomes. The distribution of *trans*:*cis* ratios over the genome identify regions of local and interchromosomal interactions. The high degree of collinearity between the new black raspberry assembly and the strawberry genome validates the quality of our assembly on both the scaffold and chromosome scale^25^.

The Hi-C chromatin conformation capture technology has both improved our understanding of genome packaging and facilitated the assembly of physical maps. An increasing number of studies have used the chromosome conformation capture technique to achieve high resolution chromosome-scale assemblies^19,26,27^. Here we have clearly demonstrated that Hi-C analysis has vastly improved the black raspberry genome assembly of ORUS 4115-3, yielding a N50 contig size for the proximity-guided assembly of 31,759,000 bp versus the N50 scaffold size of 48,488 bp for the previously assembled genome of VanBuren et al.^16^ Furthermore, our Rubus_occidentalis_v1 assembly showed a high degree of alignment with genetic maps based on GBS data, demonstrating the capability of Hi-C to correctly organize scaffolds into pseudo-chromosomes.

Despite some errors in the order of GBS markers, the majority of the genetic markers tested in this study were collinear with the physical map obtained using Hi-C. The misplaced markers could be due to the observed variable recombination rates across the genome, which often depends on GC content as well as the relative positions of markers compared to centromeres and telomeres^28^. It has been observed previously that eukaryotic genomes have regions of high and low frequency of recombination, making it difficult to establish a direct correlation between physical and genetic maps^29^. For example, Chen et al.^30^, found supressed recombination in centromeric regions and on the short arms of chromosomes 4 and 10 in rice genome.

Consistent with this work, many studies have reported inconsistencies in marker’s orders of genetic and physical maps. For example, Dewan et al.^31^, had identified that ~11% of genetic markers from chromosomes 3 and 21 in human genome were inconsistent with the physical map. Similarly, a comparison between a soybean sequence-based physical map and a genetic map, built from a cross between *G*. *max* and *G*. *soja*, revealed huge discrepancies in the markers orders of 26 genomic regions^32^. These anomalies were found to be due to the errors in genome assembly^32^. Furthermore, the evidence that genetic markers are often located at different physical position is highlighted by Bustamante et al.^33^ in barley. In this study, the authors investigated the positions and orders of genetically identified markers of chromosome 3H with a physical map, derived from fingerprinted BAC contigs. Their study showed that long genetic distances at subterminal chromosomal regions were translated into short physical distances indicating the presence of hot spots for recombination at distal regions of chromosome 3H.

Each eukaryotic chromosome has a centromere composed of highly repeated DNA sequences involved in the control of chromosome separation during meiosis. Genetic loci located near centromeres are difficult to map, due to supressed recombination frequencies at or near centromeres. Presumably, this prevents chromosome breakage, chromosome loss, and disruption in pericentric sister chromatid cohesion^34^. The complex structures together with the difficulty in distinguishing centromere repeats from the flanking repeats make the identification of centromere locations a daunting task^35,36^. For this reason, we used a combination of genetic maps, sequence search and chromosome interaction patterns derived from the Hi-C data to identify the raspberry centromere locations. We found that the centromere positions predicted by low recombination frequencies corresponded closely with those predicted by mapping the 303 bp black raspberry centromere sequence to the seven pseudo-chromosomes of the genome assembly. It should be noted that the 303 bp centromere sequence mapped to two separate locations, 4.831 and 12.686 Mb on chromosome 6, which could have been be due to errors in the genome assembly. To identify the correct position of the chromosome 6 centromere we investigated the flanking regions around the two mapped locations and compared them with the flanking sequences of the other six centromeric positions identified. This comparison suggests that the correct position of the centromere of chromosome 6 is 4.83 Mb rather than 12.686 Mb. Furthermore, chromosome 1 may has a cold spots for recombination at 22 Mb.

We also exploited the power of Hi-C analysis to identify genomic regions involved in local intrachromosomal and long-range interchromosomal interactions (Fig. 4). This information could be of significant value for gene expression studies in the future, because interchromosomal interactions can facilitate the identification of regulatory elements such as promoters and enhancers that interact with each other during chromatin folding to regulate gene expression^37^. The number of interactions occurring between genomic regions of same chromosome (*cis* interactions) is higher that the numbers occurring between the elements located on different chromosomes (*trans* interactions). Interestingly, Hi-C heat map shows low intensity and discontinuous signals along the diagonal of chromosome 6 (Fig. 1a). This may represent the hetro-chromatin regions actively involved in *trans* interactions rather than *cis* interactions (also indicated by red bars in Fig. 4).

## Conclusion

We present a high-quality reference genome for black raspberry with significantly longer scaffolds than the previously published version^16^. Our PGA-based *Rubus* genome will provide assistance in improving genetic maps for both diploid and polyploid species of the *Rosoideae*. This will accelerate the identification of DNA markers linked to desired traits thus facilitating the cost-effective and efficient development of new cultivars with these traits. Furthermore we have located the positions of each of the centromeres and mapped chromatin domains with *cis* and *trans* interactions, providing new knowledge that will have significant importance in developing an understanding of genome organization in black raspberry.

Our results provide a significant step towards improved solutions for map-based cloning of genes controlling crucial traits such as pest and disease resistance, plant architecture, and fruit form and quality.

## Electronic supplementary material

Supplementary table 1

Supplementary Table S2

Supplementary Table S3

Supplementary Figure S1

Supplementary Figure S2

Supplementary Figure S3

Supplementary Figure S4

Supplementary Figure S5

Supplementary Figure S6

Supplementary Figure S7

Supplementary Figure S8

Supplementary Figure S9
