# Supplementary material for: Chromosome-scale scaffolding of the black raspberry (Rubus occidentalis L.) genome based on chromatin interaction data
Source: Hortic Res. 2018 Feb 7;5:8. doi: 10.1038/s41438-017-0013-y (PMC5802725; doi:10.1038/s41438-017-0013-y)
Supplement: Supplementary file 2 — Supplementary Table S2 [file 41438_2017_13_MOESM2_ESM.docx]

| ORUS 4153_1 |  |  |  |  |  |  |  |
| --- | --- | --- | --- | --- | --- | --- | --- |
| Linkage map | Physical map |  | Linkage map | Physical map |  | Linkage map | Physical map |
| BraspberryS0278_218184 | BraspberryS0804_55106 | chr4 | BraspberryS0037_479829 | BraspberryS0037_479829 | chr6 | BraspberryS1797_556 | BraspberryS0380_62888 |
| BraspberryS0721_59214 | BraspberryS0929_43718 |  | BraspberryS0440_123121 | BraspberryS0440_123121 |  | BraspberryS0447_61626 | BraspberryS0114_40714 |
| BraspberryS0921_19756 | BraspberryS0648_18748 |  | BraspberryS0357_177550 | BraspberryS0357_177550 |  | BraspberryS0394_124946 | BraspberryS0252_232489 |
| BraspberryS0929_43718 | BraspberryS0278_218184 |  | BraspberryS0288_52689 | BraspberryS0288_52689 |  | BraspberryS0977_26034 | BraspberryS0087_64177 |
| BraspberryS0648_18748 | BraspberryS0010_617269 |  | BraspberryS0030_582093 | BraspberryS0630_40278 |  | BraspberryS0099_104800 | BraspberryS0431_37187 |
| BraspberryS0804_55106 | BraspberryS0088_93014 |  | BraspberryS0327_60426 | BraspberryS0174_269589 |  | BraspberryS0140_40577 | BraspberryS0047_428315 |
| BraspberryS0010_617269 | BraspberryS0097_272432 |  | BraspberryS0485_41160 | BraspberryS0030_582093 |  | BraspberryS0122_74787 | BraspberryS0487_120066 |
| BraspberryS0088_93014 | BraspberryS0154_190648 |  | BraspberryS0258_212619 | BraspberryS0327_60426 |  | BraspberryS0187_259241 | BraspberryS0671_62748 |
| BraspberryS0097_272432 | BraspberryS0081_168310 |  | BraspberryS0174_269589 | BraspberryS0258_212619 |  | BraspberryS0196_140013 | BraspberryS0394_124946 |
| BraspberryS0154_190648 | BraspberryS0015_274316 |  | BraspberryS0109_307463 | BraspberryS0502_126385 |  | BraspberryS0684_63660 | BraspberryS0215_218295 |
| BraspberryS0081_168310 | BraspberryS0721_59214 |  | BraspberryS0364_80657 | BraspberryS0537_5882 |  | BraspberryS0099_32802 | BraspberryS0977_26034 |
| BraspberryS0015_274316 | BraspberryS0921_19756 |  | BraspberryS0502_126385 | BraspberryS0109_307463 |  | BraspberryS0525_75530 | BraspberryS0041_43599 |
| BraspberryS003_792848 | BraspberryS0335_26380 |  | BraspberryS0314_148478 | BraspberryS0529_27629 |  | BraspberryS0474_45156 | BraspberryS0316_186017 |
| BraspberryS0134_217734 | BraspberryS0070_150228 |  | BraspberryS0630_40278 | BraspberryS0469_61140 |  | BraspberryS0275_97574 | BraspberryS0144_116431 |
| BraspberryS0335_26380 | BraspberryS0020_736564 |  | BraspberryS0537_5882 | BraspberryS0010_1249211 |  | BraspberryS0079_351594 | BraspberryS0024_348547 |
| BraspberryS0070_150228 |  |  | BraspberryS0529_27629 | BraspberryS0364_80657 |  | BraspberryS0065_461926 | BraspberryS0064_165666 |
| BraspberryS0020_736564 |  |  | BraspberryS0161_355312 | BraspberryS0161_355312 |  | BraspberryS0033_584984 | BraspberryS0028_107186 |
| BraspberryS0007_295570 | BraspberryS0010_66813 |  | BraspberryS0010_1249211 | BraspberryS0235_147689 |  | BraspberryS0457_17312 | BraspberryS0842_37425 |
| BraspberryS0002_372319 | BraspberryS0053_419253 |  | BraspberryS0235_147689 | BraspberryS0494_12849 |  | BraspberryS00214_34656 | BraspberryS0977_26034 |
| BraspberryS0017_207928 | BraspberryS0241_119603 |  | BraspberryS0469_61140 | BraspberryS0653_21318 |  | Ro4532_BraspberryS0013 |  |
| BraspberryS0004_725978 | BraspberryS0419_74798 |  | BraspberryS0494_12849 | BraspberryS0454_132548 |  | BraspberryS0063_573175 |  |
| BraspberryS0062_439154 | BraspberryS0134_74067 |  | BraspberryS1174_19186 | BraspberryS1174_19186 |  | BraspberryS0013_940743 |  |
| BraspberryS0014_877960 | BraspberryS0458_3294 |  | BraspberryS0653_21318 | BraspberryS0120_199903 |  | BraspberryS84_301461 | BraspberryS482_22825 |
| BraspberryS0468_92150 | BraspberryS0348_11401 |  | Ro5378_BraspberryS400 |  |  | BraspberryS34_759158 | BraspberryS781_3964 |
| BraspberryS0032_375704 | BraspberryS0650_32526 |  | BraspberryS0454_132548 |  |  | BraspberryS55_479636 | BraspberryS158_2224 |
| BraspberryS0124_714 | BraspberryS0353_156014 |  | BraspberryS0120_199903 |  |  | BraspberryS60_604050 | BraspberryS421_63343 |
| BraspberryS0022_493758 | BraspberryS0023_120199 |  | BraspberryS0147_375410 |  |  | BraspberryS98_274616 | BraspberryS604_66577 |
| BraspberryS0077_522635 | BraspberryS0019_740061 |  | BraspberryS0026_210580 | BraspberryS0026_210580 |  | BraspberryS272_71197 | BraspberryS399_120712 |
| BraspberryS0418_91496 | BraspberryS0002_372319 |  | BraspberryS0011_701791 | BraspberryS0011_701791 |  | BraspberryS94_313488 | BraspberryS121_410272 |
| BraspberryS0294_240516 | BraspberryS0017_207928 |  | BraspberryS0005_672048 | BraspberryS0005_672048 |  | BraspberryS173_278143 | BraspberryS249_52424 |
| BraspberryS0269_65757 | BraspberryS0004_725978 |  | BraspberryS0386_162892 | BraspberryS0386_162892 |  | BraspberryS200_242671 | BraspberryS547_52503 |
| BraspberryS0295_217369 | BraspberryS0062_439154 |  | BraspberryS247_264439 | BraspberryS0222_4415 |  | BraspberryS232_134490 | BraspberryS1391_6231 |
| BraspberryS0267_154502 | BraspberryS0014_877960 | chr5 | BraspberryS138_172691 | BraspberryS0146_178483 |  | BraspberryS61_40795 | BraspberryS151_34946 |
| BraspberryS0112_10927 | BraspberryS0468_92150 |  | BraspberryS0222_4415 | BraspberryS0134_395808 |  | BraspberryS110_362170 | BraspberryS1001_19490 |
| BraspberryS0456_103577 | BraspberryS0456_103577 |  | BraspberryS085_209336 | BraspberryS0051_580471 |  | BraspberryS865_3480 | BraspberryS325_109080 |
| BraspberryS0019_740061 | BraspberryS0112_10927 |  | BraspberryS0146_178483 | BraspberryS0090_260135 |  | BraspberryS705_64391 | BraspberryS219_194825 |
| BraspberryS0217_52295 | BraspberryS0077_522635 |  | BraspberryS078_386742 | BraspberryS0038_226913 |  | BraspberryS356_106736 | BraspberryS1104_1113 |
| BraspberryS0615_66181 | BraspberryS0267_154502 |  | BraspberryS0134_395808 | BraspberryS0039_50026 |  | BraspberryS544_70128 | BraspberryS277_211346 |
| BraspberryS0241_119603 | BraspberryS0294_240516 |  | BraspberryS0051_580471 | BraspberryS0220_216348 |  | BraspberryS540_84061 | BraspberryS496_44208 |
| BraspberryS0419_74798 | BraspberryS0418_91496 |  | BraspberryS0090_260135 | BraspberryS0427_121604 |  | BraspberryS104_464720 | BraspberryS500_53440 |
| BraspberryS0134_74067 | BraspberryS0269_65757 |  | BraspberryS0038_226913 | BraspberryS0259_46181 |  | BraspberryS437_106918 | BraspberryS244_154037 |
| BraspberryS0176_295101 | BraspberryS0217_52295 |  | BraspberryS0427_121604 | BraspberryS0961_14723 |  | BraspberryS689_55838 | BraspberryS471_114555 |
| Ro17803_BraspberryS176 | BraspberryS0615_66181 |  | BraspberryS0220_216348 | BraspberryS0733_27643 |  | BraspberryS1391_6231 | BraspberryS756_65801 |
| BraspberryS0458_3294 | BraspberryS0295_217369 |  | BraspberryS0259_46181 | BraspberryS0127_265724 |  | BraspberryS287_112188 | BraspberryS681_58391 |
| BraspberryS0650_32526 | BraspberryS0124_714 |  | BraspberryS0039_50026 | BraspberryS0344_12492 | chr7 | BraspberryS482_22825 | BraspberryS212_266279 |
| BraspberryS0348_11401 | BraspberryS0022_493758 |  | BraspberryS0733_27643 | BraspberryS0961_14723 |  | BraspberryS341_24920 | BraspberryS189_282081 |
| BraspberryS0590_9650 | BraspberryS0032_375704 |  | BraspberryS0344_12492 | BraspberryS1313_1723 |  | BraspberryS135_1836 | BraspberryS337_42274 |
| BraspberryS0023_120199 | BraspberryS0590_9650 |  | BraspberryS0127_265724 | BraspberryS0530_106983 |  | BraspberryS651_61319 | BraspberryS135_1836 |
| BraspberryS0353_156014 | BraspberryS0176_295101 |  | BraspberryS1127_16040 | BraspberryS0654_42261 |  | BraspberryS577_10160 | BraspberryS287_112188 |
| BraspberryS0010_66813 |  |  | BraspberryS0530_106983 | BraspberryS0961_14723 |  | BraspberryS337_42274 | BraspberryS495_85717 |
| BraspberryS0053_419253 |  |  | BraspberryS1313_1723 | BraspberryS0776_33747 |  | BraspberryS500_53440 | BraspberryS110_362170 |
| BraspberryS0026_4209 | BraspberryS0139_144731 |  | BraspberryS0395_59407 | BraspberryS0505_61312 |  | BraspberryS277_211346 | BraspberryS577_10160 |
| BraspberryS0031_314682 | BraspberryS0073_33754 |  | BraspberryS0654_42261 | BraspberryS0961_14723 |  | BraspberryS756_65801 | BraspberryS689_55838 |
| BraspberryS0021_213189 | BraspberryS0095_127410 |  | BraspberryS0776_33747 | BraspberryS0395_59407 |  | BraspberryS399_120712 | BraspberryS544_70128 |
| BraspberryS0118_113626 | BraspberryS0142_375833 |  | BraspberryS0117_462560 | BraspberryS0117_462560 |  | BraspberryS681_58391 | BraspberryS356_106736 |
| BraspberryS0209_270566 | BraspberryS0029_168757 |  | BraspberryS0505_61312 | BraspberryS0102_135460 |  | BraspberryS475_16395 | BraspberryS104_464720 |
| BraspberryS0156_147409 | BraspberryS0107_124243 |  | BraspberryS0102_135460 | BraspberryS0402_61441 |  | BraspberryS471_114555 | BraspberryS540_84061 |
| BraspberryS0167_271810 | BraspberryS0111_349602 |  | BraspberryS0402_61441 | BraspberryS0387_8229 |  | BraspberryS495_85717 | BraspberryS705_64391 |
| BraspberryS0076_223569 | BraspberryS0113_308895 |  | BraspberryS0059_190044 | BraspberryS0059_190044 |  | BraspberryS1104_1113 | BraspberryS651_61319 |
| BraspberryS0168_55970 | BraspberryS0045_71463 |  | BraspberryS0387_8229 | BraspberryS1127_16040 |  | BraspberryS496_44208 | BraspberryS61_40795 |
| BraspberryS0231_29430 | BraspberryS0080_151911 |  | BraspberryS0961_14723 |  |  | BraspberryS244_154037 | BraspberryS272_71197 |
| BraspberryS0052_429297 | BraspberryS0194_27933 |  | BraspberryS01029_12169 |  |  | BraspberryS1001_19490 | BraspberryS173_278143 |
| BraspberryS00261_153798 | BraspberryS0409_72630 |  | BraspberryS0047_428315 | BraspberryS0063_573175 |  | BraspberryS212_266279 | BraspberryS94_313488 |
| BraspberryS0408_156553 | BraspberryS0231_29430 |  | BraspberryS0083_31915 | BraspberryS0083_31915 |  | BraspberryS604_66577 | BraspberryS200_242671 |
| BraspberryS0409_72630 | BraspberryS0168_55970 |  | BraspberryS0487_120066 | BraspberryS0047_428315 |  | BraspberryS781_3964 | BraspberryS232_134490 |
| BraspberryS0279_96154 | BraspberryS0052_429297 |  | BraspberryS0144_116431 | BraspberryS0033_584984 |  | BraspberryS189_282081 | BraspberryS437_106918 |
| BraspberryS0355_30318 | BraspberryS0076_223569 |  | BraspberryS0024_348547 | BraspberryS0477_117436 |  | BraspberryS547_52503 | BraspberryS60_604050 |
| BraspberryS0194_27933 | BraspberryS0167_271810 | chr6 | BraspberryS0671_62748 | BraspberryS1797_556 |  | BraspberryS249_52424 | BraspberryS98_274616 |
| BraspberryS0226_285133 | BraspberryS0156_147409 |  | BraspberryS0064_165666 | BraspberryS0684_63660 |  | BraspberryS121_410272 | BraspberryS34_759158 |
| BraspberryS0080_151911 | BraspberryS0209_270566 |  | BraspberryS0028_107186 | BraspberryS0447_61626 |  | BraspberryS219_194825 | BraspberryS865_3480 |
| BraspberryS0045_71463 | BraspberryS0118_113626 |  | BraspberryS0087_64177 | BraspberryS0099_104800 |  | BraspberryS325_109080 | BraspberryS341_24920 |
| BraspberryS0111_349602 | BraspberryS0021_213189 |  | BraspberryS0252_232489 | BraspberryS0065_461926 |  | BraspberryS151_34946 | BraspberryS287_112188 |
| BraspberryS0113_308895 | BraspberryS0031_314682 |  | BraspberryS0114_40714 | BraspberryS0079_351594 |  | BraspberryS421_63343 | BraspberryS55_479636 |
| BraspberryS0107_124243 | BraspberryS0408_156553 |  | BraspberryS0380_62888 | BraspberryS0275_97574 |  | BraspberryS158_2224 |  |
| BraspberryS0029_168757 |  |  | BraspberryS0316_186017 | BraspberryS0196_140013 |  |  |  |
| BraspberryS0139_144731 |  |  | BraspberryS0431_37187 | BraspberryS0684_63660 |  |  |  |
| BraspberryS0095_127410 |  |  | BraspberryS0842_37425 | BraspberryS0474_45156 |  |  |  |
| BraspberryS0142_375833 |  |  | BraspberryS0041_43599 | BraspberryS0122_74787 |  |  |  |
| Braspberry00S46_579890 |  |  | BraspberryS0371_49180 | BraspberryS0187_259241 |  |  |  |
| BraspberryS0073_33754 |  |  | BraspberryS0376_162362 | BraspberryS0431_37187 |  |  |  |
| BraspberryS0462_77258 | BraspberryS0001_8641 |  | Ro11481_BraspberryS0328 | BraspberryS0525_75530 |  |  |  |
| BraspberryS0001_2397101 | BraspberryS0153_124032 |  | BraspberryS0215_218295 | BraspberryS0140_40577 |  |  |  |
| BraspberryS0153_124032 | BraspberryS0462_77258 |  | BraspberryS0818_21981 | BraspberryS0818_21981 |  |  |  |
| BraspberryS0001_8641 | BraspberryS0485_41160 |  | BraspberrySQ07-4_D05_RLG6 | BraspberryS0371_49180 |  |  |  |
| BraspberryS0391_173965 | BraspberryS0391_173965 |  | BraspberryS0477_117436 | BraspberryS0376_162362 |  |  |  |
