# Supplementary material for: Chromosome-scale scaffolding of the black raspberry (Rubus occidentalis L.) genome based on chromatin interaction data
Source: Hortic Res. 2018 Feb 7;5:8. doi: 10.1038/s41438-017-0013-y (PMC5802725; doi:10.1038/s41438-017-0013-y)
Supplement: Supplementary file 3 — Supplementary Table S3 [file 41438_2017_13_MOESM3_ESM.docx]

|  | **ORUS 3021_1** |  |  |  |  |  |
| --- | --- | --- | --- | --- | --- | --- |
|  | Linkage map | Physical map |  | Linkage map | Physical map | |
| chr1 | BraspberryS0472_89825 | BraspberryS0804_55106 | chr5 | BraspberryS0026_210580 | BraspberryS0026_210580 | |
|  | BraspberryS0921_19756 | BraspberryS0143_181231 |  | BraspberryS0011_701791 | BraspberryS0011_701791 | |
|  | BraspberryS0929_43718 | BraspberryS0014_952525 |  | BraspberryS0005_672048 | BraspberryS0005_672048 | |
|  | BraspberryS0798_31088 | BraspberryS0426_20245 |  | BraspberryS0386_162892 | BraspberryS0386_162892 | |
|  | BraspberryS0142_215410 | BraspberryS0798_31088 |  | BraspberryS247_264439 | BraspberryS0222_4415 | |
|  | BraspberryS0414_7874 | BraspberryS0929_43718 |  | BraspberryS138_172691 | BraspberryS0146_178483 | |
|  | BraspberryS0999_22487 | BraspberryS0648_18748 |  | BraspberryS0222_4415 | BraspberryS0134_395808 | |
|  | BraspberryS0648_18748 | BraspberryS0143_181231 |  | BraspberryS085_209336 | BraspberryS0051_580471 | |
|  | BraspberryS0426_20245 | BraspberryS0234_258231 |  | BraspberryS0146_178483 | BraspberryS0090_260135 | |
|  | BraspberryS0014_952525 | BraspberryS0081_168310 |  | BraspberryS078_386742 | BraspberryS0038_226913 | |
|  | BraspberryS0721_59214 | BraspberryS0075_381030 |  | BraspberryS0134_395808 | BraspberryS0039_50026 | |
|  | BraspberryS1043_181231 | BraspberryS0565_20930 |  | BraspberryS0051_580471 | BraspberryS0220_216348 | |
|  | BraspberryS0565_20930 | BraspberryS0721_59214 |  | BraspberryS0090_260135 | BraspberryS0427_121604 | |
|  | BraspberryS0804_55106 | BraspberryS0042_237147 |  | BraspberryS0038_226913 | BraspberryS0259_46181 | |
|  | BraspberryS0088_93014 | BraspberryS0020_127876 |  | BraspberryS0427_121604 | BraspberryS0961_14723 | |
|  | BraspberryS0234_258231 |  |  | BraspberryS0220_216348 | BraspberryS0733_27643 | |
|  | BraspberryS0097_272432 |  |  | BraspberryS0259_46181 | BraspberryS0127_265724 | |
|  | BraspberryS0081_168310 |  |  | BraspberryS0039_50026 | BraspberryS0344_12492 | |
|  | BraspberryS0075_381030 |  |  | BraspberryS0733_27643 | BraspberryS0961_14723 | |
|  | BraspberryS0042_237147 |  |  | BraspberryS0344_12492 | BraspberryS1313_1723 | |
|  | BraspberryS0020_127876 |  |  | BraspberryS0127_265724 | BraspberryS0530_106983 | |
| chr2 | BraspberryS0007_379506 | BraspberryS0169_39974 |  | BraspberryS1127_16040 | BraspberryS0654_42261 | |
|  | BraspberryS0071_420905 | BraspberryS0727_30152 |  | BraspberryS0530_106983 | BraspberryS0961_14723 | |
|  | BraspberryS0002_650888 | BraspberryS0134_83955 |  | BraspberryS1313_1723 | BraspberryS0776_33747 | |
|  | BraspberryS0002_739570 | BraspberryS0714_73912 |  | BraspberryS0395_59407 | BraspberryS0505_61312 | |
|  | BraspberryS0004_44081 | BraspberryS0449_31741 |  | BraspberryS0654_42261 | BraspberryS0961_14723 | |
|  | BraspberryS0004_142584 | BraspberryS0149_322226 |  | BraspberryS0776_33747 | BraspberryS0395_59407 | |
|  | BraspberryS0014_44081 | BraspberryS0007_379506 |  | BraspberryS0117_462560 | BraspberryS0117_462560 | |
|  | BraspberryS0294_233966 | BraspberryS0002_650888 |  | BraspberryS0505_61312 | BraspberryS0102_135460 | |
|  | BraspberryS0295_217369 | BraspberryS0295_217369 |  | BraspberryS0102_135460 | BraspberryS0402_61441 | |
|  | BraspberryS0152_94166 | BraspberryS0112_10927 |  | BraspberryS0402_61441 | BraspberryS0387_8229 | |
|  | BraspberryS0077_150939 | BraspberryS0152_94166 |  | BraspberryS0059_190044 | BraspberryS0059_190044 | |
|  | BraspberryS0615_66181 | BraspberryS0294_233966 |  | BraspberryS0387_8229 | BraspberryS1127_16040 | |
|  | BraspberryS0217_52295 | BraspberryS0217_52295 |  | BraspberryS0961_14723 |  |  |
|  | BraspberryS0332_25238 | BraspberryS0590_9650 |  | BraspberryS01029_12169 |  |  |
|  | BraspberryS0112_10927 |  | chr6 | BraspberryS0047_293993 | BraspberryS0063_573175 | |
|  | BraspberryS0134_83955 |  |  | BraspberryS0155_368623 | BraspberryS0214_34663 | |
|  | BraspberryS0714_73912 |  |  | BraspberryS0028_64542 | BraspberryS1158_2328 | |
|  | BraspberryS0590_9650 |  |  | BraspberryS0087_253649 | BraspberryS0238_33526 | |
|  | BraspberryS0169_39974 |  |  | BraspberryS0256_68947 | BraspberryS1158_2328 | |
|  | BraspberryS0449_31741 |  |  | BraspberryS0266_83737 | BraspberryS0266_83737 | |
|  | BraspberryS0149_322226 |  |  | BraspberryS0780_47083 | BraspberryS0780_47083 | |
|  | BraspberryS0727_30152 |  |  | BraspberryS0187_259241 | BraspberryS0049_534988 | |
| chr3 | BraspberryS0068:477340 | BraspberryS0202:203089 |  | BraspberryS0238_33526 | BraspberryS0331_120820 | |
|  | BraspberryS0119:192278 | BraspberryS0073_33754 |  | BraspberryS0492_56949 | BraspberryS0065_461926 | |
|  | BraspberryS0026:335827 | BraspberryS0095_41560 |  | BraspberryS1158_2328 | BraspberryS0136_346904 | |
|  | BraspberryS0031:314682 | BraspberryS0046_579890 |  | BraspberryS0122_230445 | BraspberryS0165_315500 | |
|  | BraspberryS0314:179125 | BraspberryS0029:4,211,544 |  | BraspberryS0196_140013 | BraspberryS0260_4228 | |
|  | BraspberryS0021:897073 | BraspberryS0075:81406 |  | BraspberryS0474_19898 | BraspberryS0474_19898 | |
|  | BraspberryS0209_265706 | BraspberryS0107:118411 |  | BraspberryS0260_4228 | BraspberryS0196_140013 | |
|  | BraspberryS0156:263195 | BraspberryS0177:269209 |  | BraspberryS0165_315500 | BraspberryS0122_230445 | |
|  | BraspberryS0175:120669 | BraspberryS0095:41560 |  | BraspberryS0136_346904 | BraspberryS0187_259241 | |
|  | BraspberryS0201:50091 | BraspberryS0142:7482 |  | BraspberryS0065_461926 | BraspberryS0492_56949 | |
|  | BraspberryS0167:305583 | BraspberryS0006:272056 |  | BraspberryS0331_120820 | BraspberryS0087_253649 | |
|  | BraspberryS0025:886295 | BraspberryS0040:488292 |  | BraspberryS0049_534988 | BraspberryS0047_293993 | |
|  | BraspberryS0380:138595 | BraspberryS0139:144731 |  | BraspberryS0214_34663 | BraspberryS0028_64542 | |
|  | BraspberryS0168:55970 | BraspberryS0113:305041 |  | BraspberryS0063_573175 | BraspberryS0155_368623 | |
|  | BraspberryS0035:190714 | BraspberryS0080:303146 | chr7 | BraspberryS0129_153488 | BraspberryS0475_16395 | |
|  | BraspberryS0052:429297 | BraspberryS0409:72552 |  | BraspberryS0631_78691 | BraspberryS1089_19238 | |
|  | BraspberryS0115:211947 | BraspberryS0318:155707 |  | BraspberryS043_656815 | BraspberryS0329_82033 | |
|  | BraspberryS0086:272676 | BraspberryS0233:65965 |  | BraspberryS0272_158581 | BraspberryS0121_410272 | |
|  | BraspberryS0318:155707 | BraspberryS0035:190714 |  | BraspberryS0251_17084 | BraspberryS0865_3480 | |
|  | BraspberryS0409:72552 | BraspberryS0202:203089 |  | BraspberryS0232-134490 | BraspberryS0799_55672 | |
|  | BraspberryS0202:203089 | BraspberryS0168:55970 |  | BraspberryS0200_237792 | BraspberryS0595_84360 | |
|  | BraspberryS0045:71463 | BraspberryS0052:429297 |  | Braspberry0S148-61171 | BraspberryS0604_66577 | |
|  | BraspberryS0239:140073 | BraspberryS0167:305583 |  | BraspberryS0865-3480 | BraspberryS0205_279114 | |
|  | BraspberryS0080:303146 | BraspberryS0201:50091 |  | BraspberryS0115-45212 | BraspberryS0547_52503 | |
|  | BraspberryS0233:65965 | BraspberryS0175:120669 |  | BraspberryS0110-263544 | BraspberryS0472_89891 | |
|  | BraspberryS0113:305041 | BraspberryS0209_265706 |  | BraspberryS0061_295208 | BraspberryS0325_109080 | |
|  | BraspberryS0027:903196 | BraspberryS0026:335827 |  | BraspberryS0205_279114 | BraspberryS0500_45749 | |
|  | BraspberryS0040:488292 | BraspberryS0314:179125 |  | BraspberryS0472_89891 | BraspberryS0219_198144 | |
|  | BraspberryS0006:272056 | BraspberryS0068:477340 |  | BraspberryS0705_64391 | BraspberryS0681_58391 | |
|  | BraspberryS0177:269209 |  |  | BraspberryS1212_14404 | BraspberryS0337_42274 | |
|  | BraspberryS0107:118411 |  |  | BraspberryS0384_49752 | BraspberryS0189_282081 | |
|  | BraspberryS0959:Ro7270 |  |  | BraspberryS0473_16767 | BraspberryS0464_41282 | |
|  | BraspberryS0029:647815 |  |  | BraspberryS0329_82033 | BraspberryS0608_26380 | |
|  | BraspberryS0075:81406 |  |  | BraspberryS0563_15061 | BraspberryS0161_75403 | |
|  | BraspberryS0139:162862 |  |  | BraspberryS0482_22825 | BraspberryS0577_10160 | |
|  | BraspberryS0334:Ro3017 |  |  | BraspberryS0341_24920 | BraspberryS0384_49752 | |
|  | BraspberryS0142:7482 |  |  | BraspberryS0464_41282 | BraspberryS0197_230674 | |
|  | BraspberryS0095:41560 |  |  | BraspberryS0021_550004 | BraspberryS0705_64391 | |
|  | BraspberryS0046:702636 |  |  | BraspberryS0197_230674 | BraspberryS0151_134836 | |
|  | BraspberryS0073:33754 |  |  | BraspberryS0651_61319 | BraspberryS0608_295208 | |
|  | BraspberryS0139:144731 |  |  | BraspberryS0681_58391 | BraspberryS0061_295208 | |
| chr4 | BraspberryS0101_278550 | BraspberryS0001_1883174 |  | BraspberryS0229_214944 | BraspberryS0651_61319 | |
|  | BraspberryS0462_77258 | BraspberryS0001_42487 |  | BraspberryS0189_282081 | BraspberryS115_45212 | |
|  | BraspberryS0001_1883174 | BraspberryS0001_2397001-2397201 | | BraspberryS0577_10160 | BraspberryS0129_153488 | |
|  | BraspberryS0001_42487 | BraspberryS0101_278550 |  | BraspberryS1034_26022 | BraspberryS0148_61171 | |
|  | BraspberryS0037_479829 | BraspberryS0485_41160 |  | BraspberryS0277_211361 | BraspberryS0110_263544 | |
|  | BraspberryS0440_123121 | BraspberryS0037_479829 |  | BraspberryS0399_120712 | BraspberryS1034_26022 | |
|  | BraspberryS0357_177604 | BraspberryS0357_177604 |  | BraspberryS0500_45749 | BraspberryS1001_19490 | |
|  | BraspberryS0288_52689 | BraspberryS0288_52689 |  | BraspberryS0608_26380 | BraspberryS1104_1113 | |
|  | BraspberryS0485_41160 | BraspberryS0225_138869 |  | BraspberryS0475_16395 | BraspberryS0473_16767 | |
|  | BraspberryS0258_212619 | BraspberryS0116_118019 |  | BraspberryS0337_126476 | BraspberryS1212_14404 | |
|  | BraspberryS0174_269589 | BraspberryS0174_269589 |  | BraspberryS495_85717 | BraspberryS0865_3480 | |
|  | BraspberryS0109_307463 | BraspberryS0258_212619 |  | BraspberryS1104_1113 | BraspberryS0341_24920 | |
|  | BraspberryS0537_5881 | BraspberryS0116_118019 |  | BraspberryS0496_44208 |  |  |
|  | BraspberryS1023_16069 | BraspberryS0109_307463 |  | BraspberryS0161_75403 |  |  |
|  | BraspberryS0529_27629 | BraspberryS0509_84964 |  | BraspberryS0595_84360 |  |  |
|  | BraspberryS0116_118019 | BraspberryS0469_61140 |  | BraspberryS1001_19490 |  |  |
|  | BraspberryS0850_14502 | BraspberryS0398_55678 |  | BraspberryS0799_55672 |  |  |
|  | BraspberryS0469_61140 | BraspberryS0509_84964 |  | BraspberryS0604_66577 |  |  |
|  | BraspberryS0653_21318 | BraspberryS0494_12860 |  | BraspberryS0547_52503 |  |  |
|  | BraspberryS1174_19186 | BraspberryS0103_169793 |  | BraspberryS0249_52424 |  |  |
|  | BraspberryS0494_12860 | BraspberryS0454_55758 |  | BraspberryS0121_410272 |  |  |
|  | BraspberryS0398_55678 | BraspberryS0253_22396 |  | BraspberryS0026_586022 |  |  |
|  | BraspberryS0103_169793 | BraspberryS0120_78654 |  | BraspberryS0219_198144 |  |  |
|  | BraspberryS0225_138869 | BraspberryS0723_48232 |  | BraspberryS0325_109080 |  |  |
|  | BraspberryS0454_55758 |  |  | BraspberryS0151_134836 |  |  |
|  | BraspberryS0253_22396 |  |  | BraspberryS1089_19238 |  |  |
|  | BraspberryS0723_48232 |  |  |  |  |  |
|  | BraspberryS0612_66375 |  |  |  |  |  |
|  | BraspberryS0120_78654 |  |  |  |  |  |
|  | BraspberryS0509_84964 |  |  |  |  |  |
