# Supplementary figures and images for: Chromosome-scale scaffolding of the black raspberry (Rubus occidentalis L.) genome based on chromatin interaction data
Source: Hortic Res. 2018 Feb 7;5:8. doi: 10.1038/s41438-017-0013-y (PMC5802725; doi:10.1038/s41438-017-0013-y)

## Slide 1
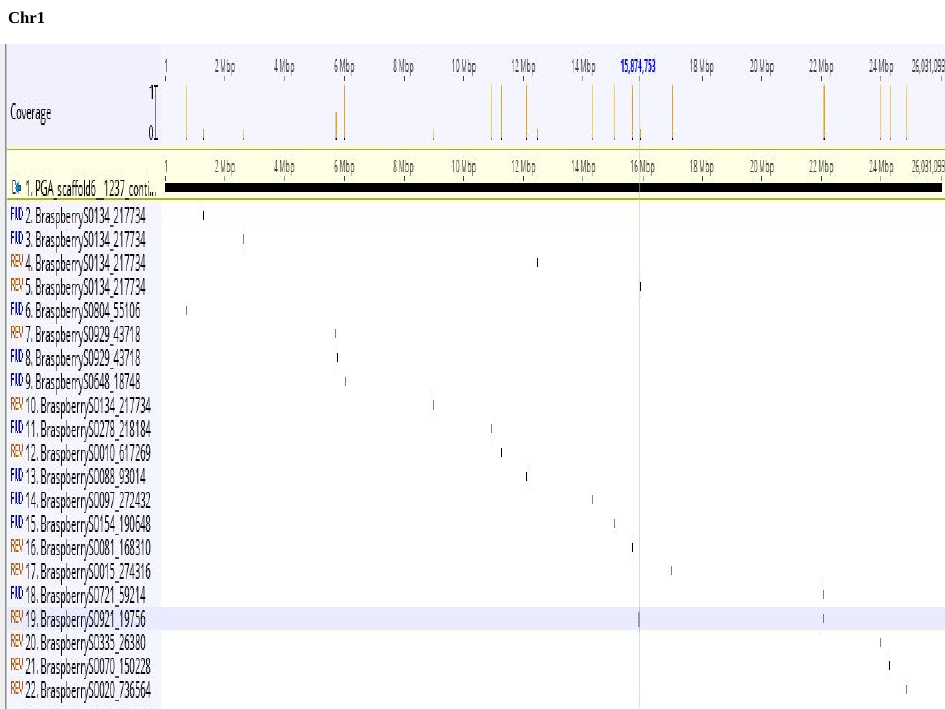

Chr1

Supplement: Supplementary file 4 — Supplementary Figure S1 [file 41438_2017_13_MOESM4_ESM.pptx]

## Slide 1
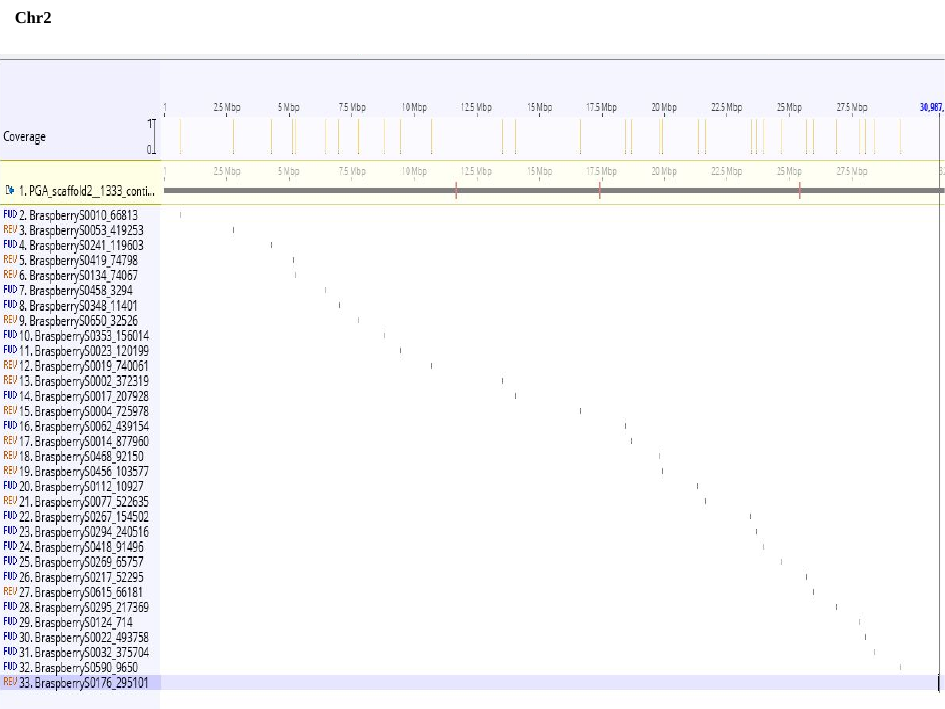

Chr2

Supplement: Supplementary file 5 — Supplementary Figure S2 [file 41438_2017_13_MOESM5_ESM.pptx]

## Slide 1
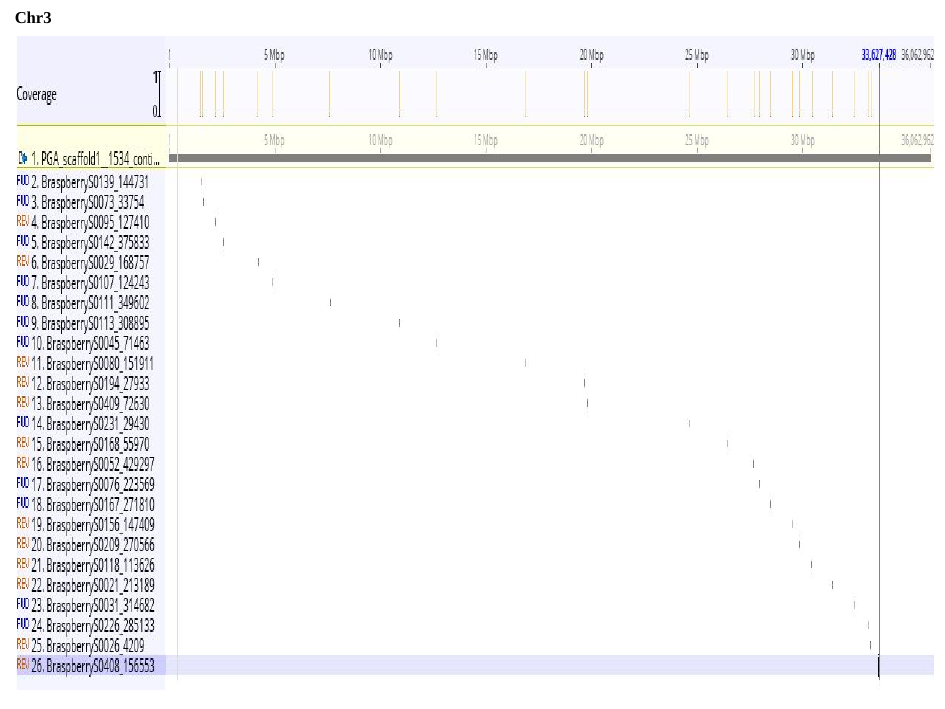

Chr3

Supplement: Supplementary file 6 — Supplementary Figure S3 [file 41438_2017_13_MOESM6_ESM.pptx]

## Slide 1
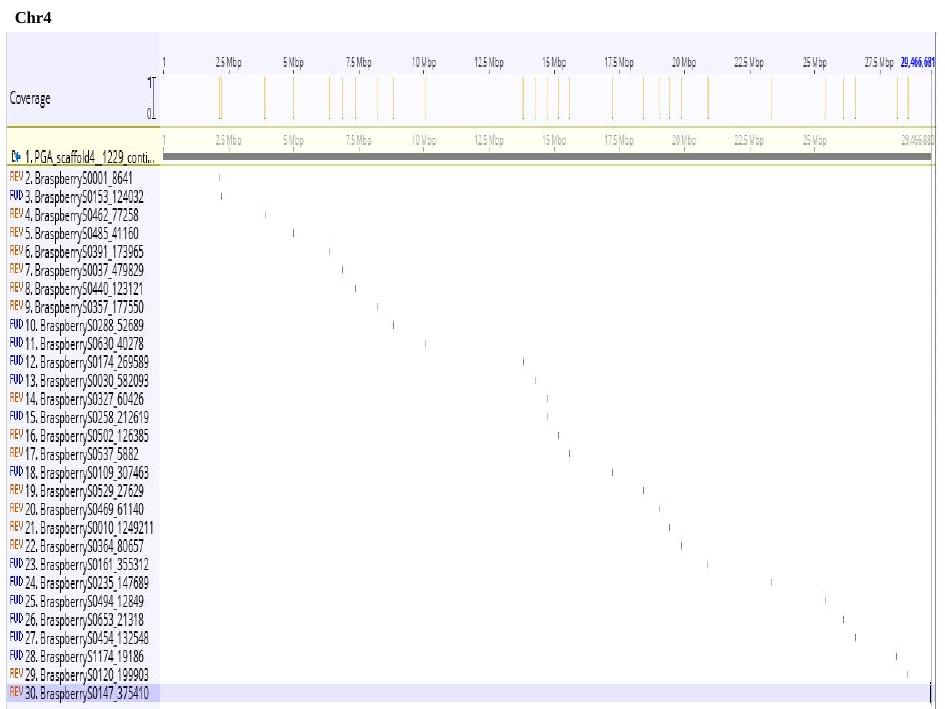

Chr4

Supplement: Supplementary file 7 — Supplementary Figure S4 [file 41438_2017_13_MOESM7_ESM.pptx]

## Slide 1
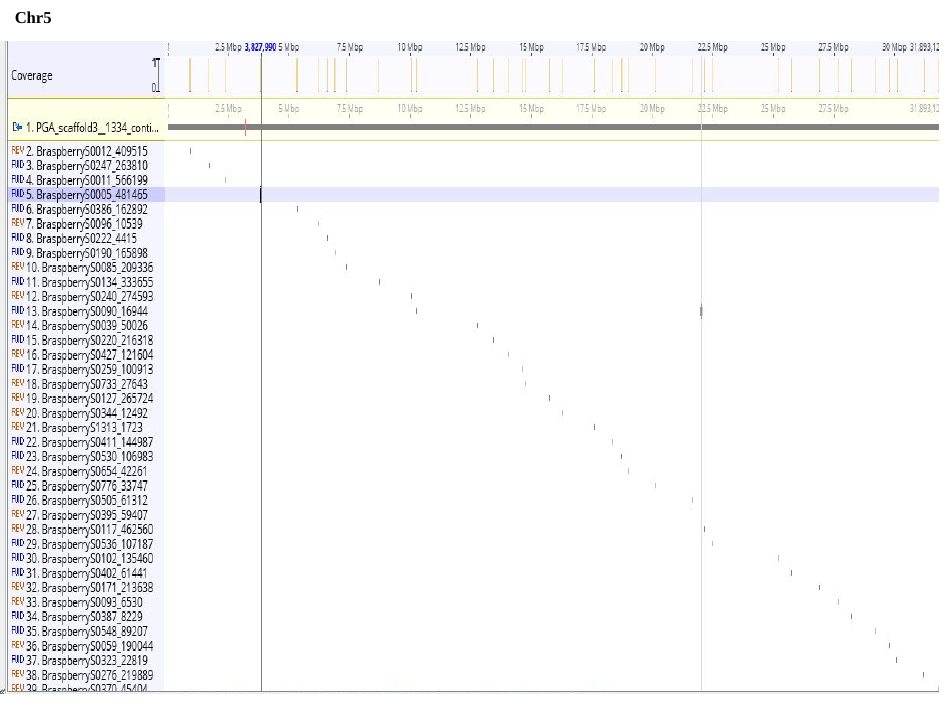

Chr5

Supplement: Supplementary file 8 — Supplementary Figure S5 [file 41438_2017_13_MOESM8_ESM.pptx]

## Slide 1
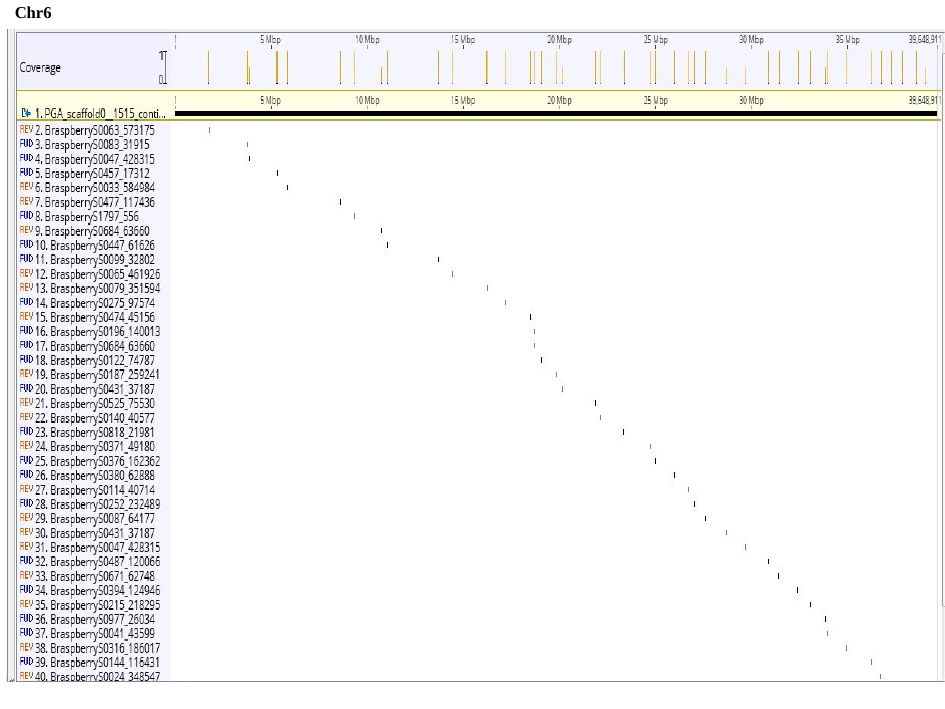

Chr6

Supplement: Supplementary file 9 — Supplementary Figure S6 [file 41438_2017_13_MOESM9_ESM.pptx]

## Slide 1
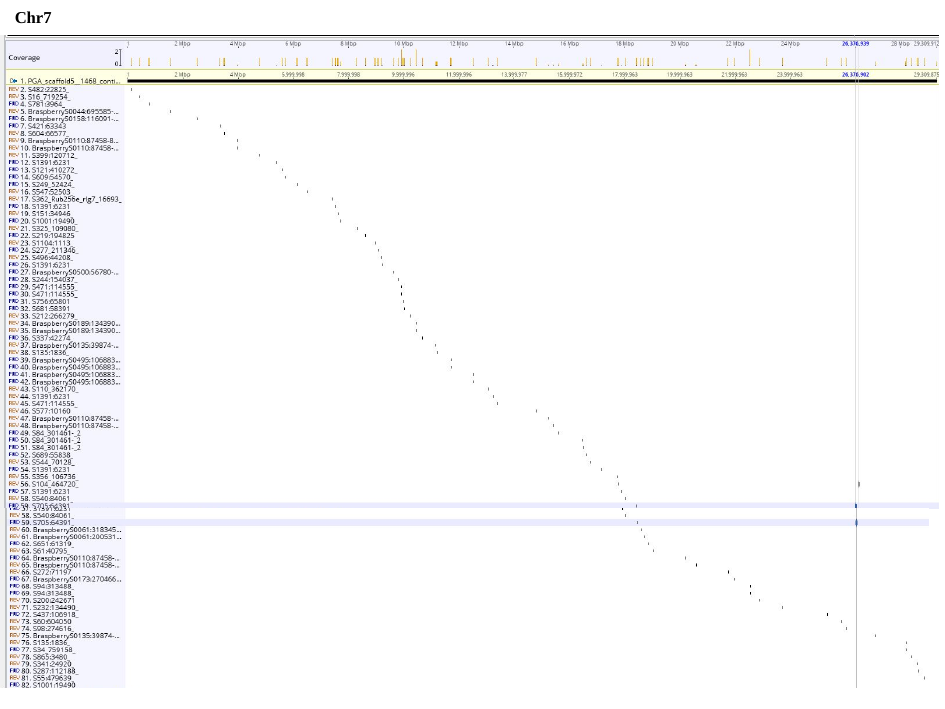

Chr7

Supplement: Supplementary file 10 — Supplementary Figure S7 [file 41438_2017_13_MOESM10_ESM.pptx]

## Slide 1
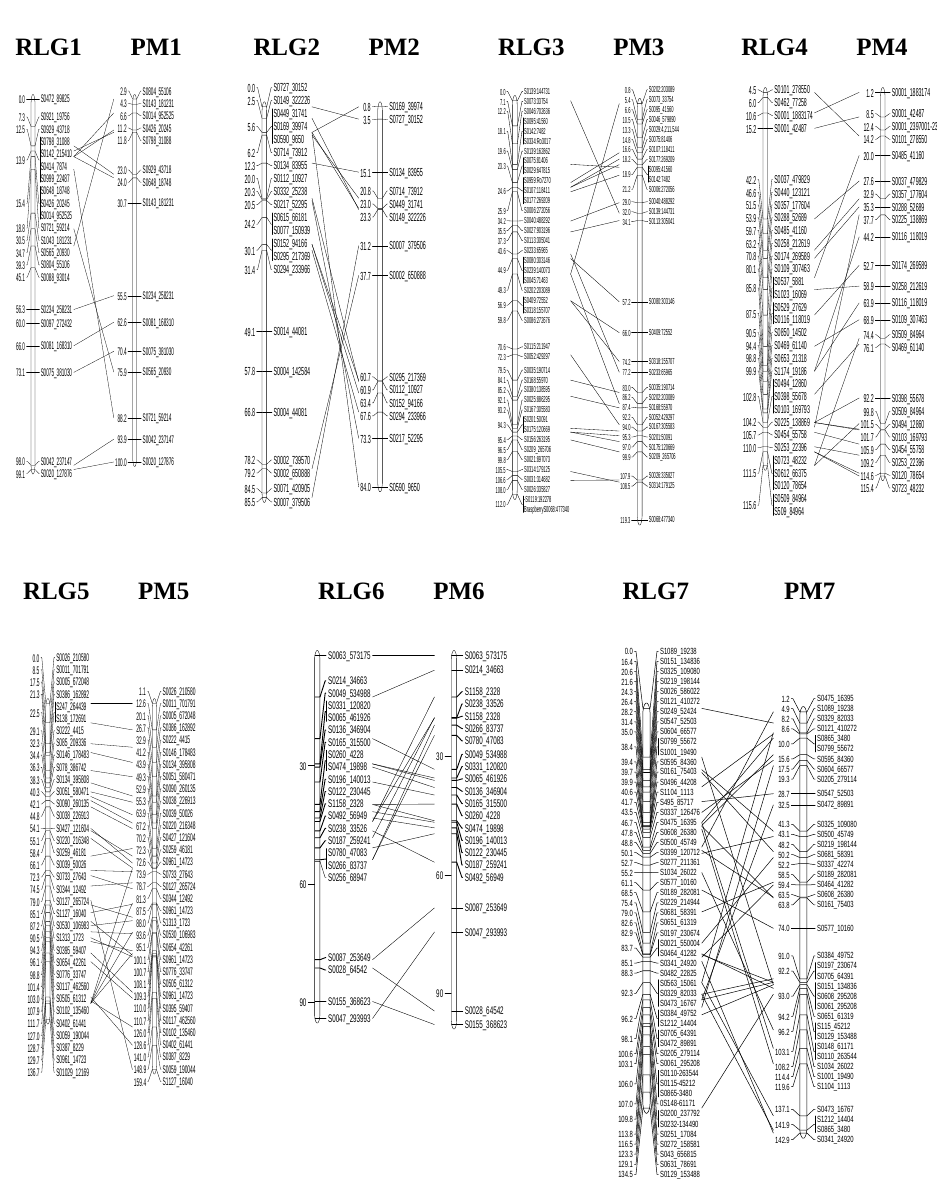

RLG1
PM1
RLG2
PM2
RLG3
PM3
RLG4
PM4
RLG5
PM5
RLG6
PM6
RLG7
PM7

Supplement: Supplementary file 11 — Supplementary Figure S8 [file 41438_2017_13_MOESM11_ESM.pptx]

## Slide 1
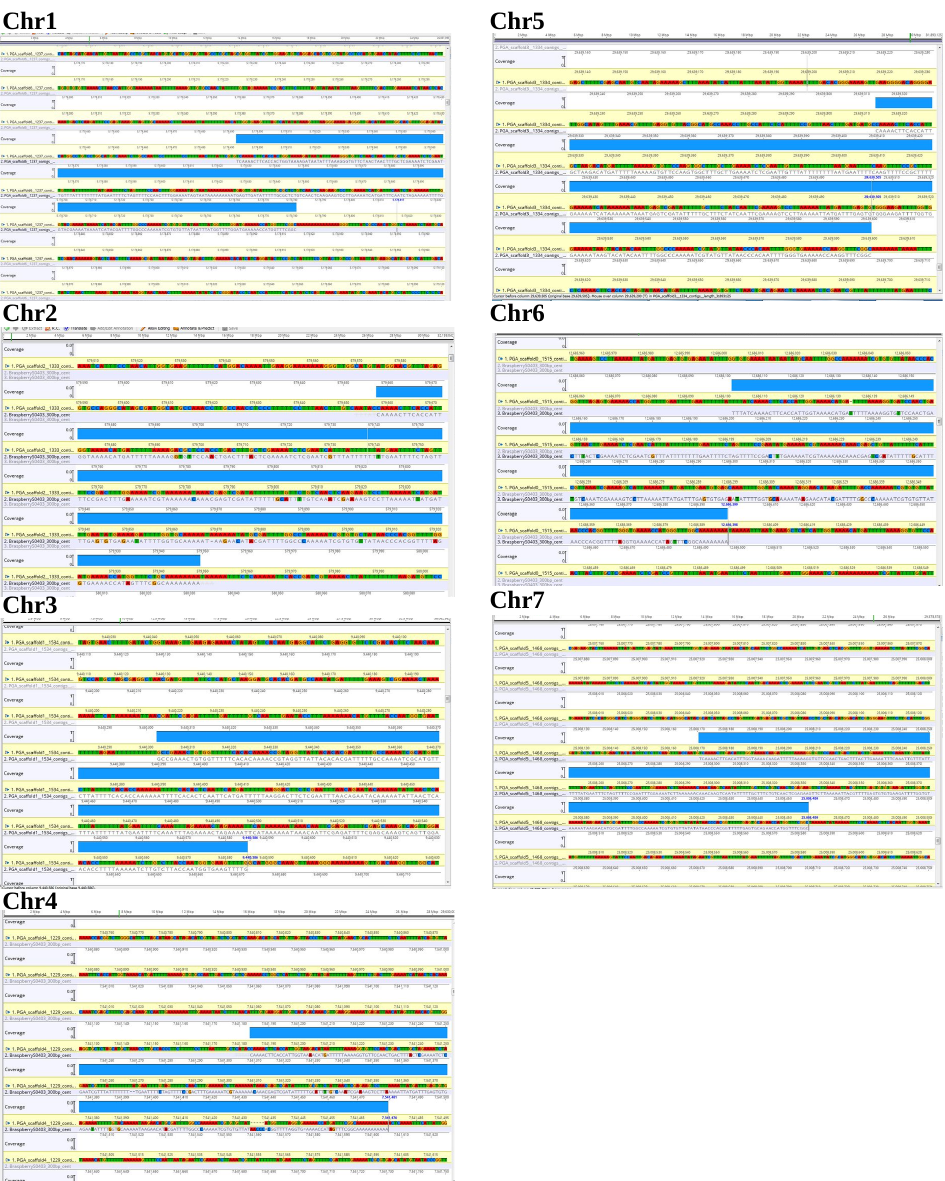

Chr1
Chr5
Chr2
Chr6
Chr7
Chr3
Chr4

Supplement: Supplementary file 12 — Supplementary Figure S9 [file 41438_2017_13_MOESM12_ESM.pptx]
